# Neutropenia after intravenous immunoglobulin therapy is associated with coronary artery lesions in children with Kawasaki disease: a case control study

**DOI:** 10.1186/s12887-018-1032-z

**Published:** 2018-02-21

**Authors:** Zhenquan Wang, Fengfeng Weng, Chen Li, Hongying Shi, Zhangke Tang, Huixian Qiu, Yue’e He, Rongzhou Wu, Maoping Chu

**Affiliations:** 1 Children’s Heart Center, the Second Affiliated Hospital and Yuying Children’s Hospital, Institute of cardiovascular development and translational medicine, Wenzhou Medical University, Wenzhou, Zhejiang 325027 China; 2 Children’s Hospital, Zhejiang University School of Medicine, Hangzhou, Zhejiang 310051 China; 3 Department of Preventive Medicine, School of Environmental Science and Public Health, Wenzhou Medical University, Wenzhou, Zhejiang 325035 China

**Keywords:** Kawasaki disease, Neutropenia, Coronary artery lesions

## Abstract

**Background:**

To evaluate differences in laboratory parameters, clinical presentation, and incidence of coronary artery lesions (CAL) between children with neutropenic and non-neutropenic Kawasaki disease (KD).

**Methods:**

All consecutive KD patients that presented to the Second Affiliated Hospital and Yuying Children’s Hospital of Wenzhou Medical University in Wenzhou, China between January 2005 and December 2015 were included in this study. Patients were divided into two groups (KD with neutropenia (NKD) and KD without neutropenia (NNKD)) based on whether or not they developed neutropenia during the course of treatment. We compared differences in clinical manifestations, laboratory parameters, and treatment protocols between groups. We also evaluated the relationship between neutropenia with immunoglobulin dosage and incidence of CAL.

**Results:**

An IVIG treatment regimen of 2 g/kg*1d was associated with a lower incidence of neutropenia compared to the 1 g/kg*2d protocol. The incidence of CAL was higher in KD patients with neutropenia than in those without. Subgroup analysis showed no difference in the incidence of CAL among the different age groups between KD patients with and without neutropenia.

**Conclusions:**

Follow up ultrasonic echocardiography should be performed in KD patients with neutropenia in order to allow for early detection of CAL and timely intervention.

## Background

Kawasaki disease (KD) is a systemic vasculitis of unknown etiology that occurs most commonly in infants and young children under 5 years old. The presenting features of KD include fever, bilateral nonexudative conjunctivitis, erythema of the lips and oral mucosa, changes in the extremities, rash, and cervical lymphadenopathy [1, 2]. KD has important cardiovascular sequelae which must be monitored and managed, the most common of which are coronary artery lesions (CAL). Intravenous immune globulin (IVIG) and aspirin are commonly used in the treatment of KD, and IVIG is particularly important due to its ability to relieve inflammation and reduce the incidence of coronary artery lesions [3].

Neutrophils play an important role in the pathogenesis of KD, as raised neutrophil levels during the course of disease have been shown to be related to the pathogenesis of KD and CAL [4]. In recent years, neutrophils have been found to be elevated in the acute phase of KD, despite a decrease in granulocyte counts and even a lack of granulocytes after treatment. The specific mechanism whereby raised neutrophil levels contribute to KD pathogenesis has not been clearly elucidated. Therefore, the aim of this study was to 1) investigate the effect of IVIG in patients with neutropenic KD (NKD) and non-neutropenic KD (NNKD); 2) compare the effects of neutropenia on laboratory markers, clinical manifestation of disease, coronary artery lesions and non-responsiveness to IVIG; and 3) study the specificity and possible mechanism of neutropenia in KD. Finally, we aimed to investigate the relationship between neutropenia with KD treatment and prognosis.

## Methods

### Subjects

We performed a retrospective medical record review of all KD inpatients from January 1, 2005 to December 31, 2015 at the Second Affiliated Hospital and Yuying Children’s Hospital of Wenzhou Medical University in Wenzhou, China. Additionally, follow-up information regarding CAL was extracted from outpatient medical records. Inclusion criteria were: (1) patients diagnosed in accordance with the Japanese KD diagnosis, (2) treated according to the clinical manifestations and ultrasonic echocardiography (UCG) results, and (3) patients with first presentation of KD [1]. We initially identified a total of 1667 (1111 male and 556 female). Patients were excluded if they had incomplete data. After applying these criteria, we included 1365 cases into the final analysis. Patients were divided into two groups according to the presence of neutropenia after IVIG treatment (NKD, 197 patients; and NNKD, 1168 patients). Among them, 539 patients received the 2 g/kg*1d program and 192 received the 1 g/kg*2d program, the rest of patients were not received IVIG or lack of sufficient information regarding IVIG treatment. All KD inpatients were initially treated with aspirin.

### Outcomes

Outcomes of interest were the timing and dose of IVIG, use of dipyridamole, laboratory parameters, clinical manifestations, and echocardiographic results. All patients were followed up for 3 months after IVIG treatment.

### Neutropenia [5]

Neutropenia is a syndrome caused by a decrease in the absolute value of peripheral blood granulocytes. Neutropenia is diagnosed based on an absolute neutrophil count less than 1.0 × 10^9^/L in children aged 2 weeks to 1 year old, or less than 1.5 × 10^9^/L in children aged over 1 year old. Agranulocytosis is defined as an absolute neutrophil count less than 0.5 × 10^9^/L.

### CAL [5]

The diagnosis of CAL is based on the following three criteria: 1) Coronary artery dilation: coronary artery diameter > 2.5 mm in children < 3 years old, > 3 mm in children 3–9 years old, and > 3.5 mm in children older than 9; as well as diameter of one segment of the coronary artery more than 1.5 times that of the adjacent segment; 2) Coronary artery aneurysm (CAA): ratio of the diameter of the coronary artery to the adjacent segment > 1.5, and diameter of the coronary artery > 4 mm. Small, medium, and giant CAAs are defined based on the coronary artery diameter: < 5 mm, 5–8 mm and > 8 mm, respectively. 3) Coronary artery stenosis and embolism: coronary artery diameter reduction, irregular and asymmetric tube wall or irregularity and interruption of the lumen of the continuous non echo area.

### Statistical analysis

Statistical analyses were performed using SPSS version 19. Measurement data are expressed by the median and the interquartile range, and the count data is represented by the number of cases and the percentage. Continuous variables were compared using Kruskal-Wallis test and categorical variables were compared using Chi-square test. Logistic regression analysis and curve fitting were used to analyze the correlation between degree of reduction in granulocytes and CAL. All tests were considered significant under the 0.05 level.

## Results

### Comparison of laboratory parameters between KD patients with and without neutropenia

Table 1 shows the laboratory parameters of children in the NKD and NNKD groups. There was a statistically significant difference between groups in pre-treatment white blood cell count (WBC), absolute neutrophil count (ANC), difference in absolute neutrophil count before and after treatment (△ANC), D-Dimer level, fibrinogen (FIB) level, and prothrombin time (PT). We found that (1) neutropenic KD patients had lower WBC and ANC levels in the acute phase after IVIG treatment (*P* = 0.028 and *P* = 0.002, respectively); (2) there was a greater reduction in ANC levels in the NKD group than the NNKD group (*P* = 0.001); and (3) D-Dimer, FIB and PT were lower in the NKD group than in the NNKD group (*P* = 0.002, *P* = 0.004, and P = 0.001, respectively).

**Table 1:** Laboratory parameters of children with KD

Laboratory parameters	NKD	NNKD	*P* value
CRP (mg/L)	70.1(32.90–105.0)	74.7(37.95–118.0)	0.147
WBC (×10^9^/L)	14.27(11.24–19.04)	15.60(11.91–19.82)	0.028
ANC1 (×10^9^/L)	0.99(0.75–1.25)	3.53(2.09–4.20)	< 0.001
ANC (×10^9^/L)	8.66(5.96–12.64)	9.89(7.02–13.67)	0.002
△ANC (×10^9^/L)	7.84(4.87–11.48)	6.49(3.57–10.17)	0.001
Hb (g/L)	109(102–115)	110(102–117)	0.268
ALT (U/L)	29.5(17.0–72.5)	32.0(17.8–90.0)	0.304
ESR (mm/h)	37.0(24.3–46.0)	35.0(26.0–45.0)	0.720
PLT (×10^9^/L)	376(299–465)	359(294–449)	0.191
ALB (g/L)	33.0(28.9–39.1)	33.3(29.1–38.4)	0.886
BNP (pg/ml)	670(319–1543)	725(305–1934)	0.528
Na (mmol/L)	136.0(134.1–137.6)	136.1(134.2–137.7)	0.433
PT (s)	13.2(12.8–13.9)	13.6(12.9–14.3)	0.001
APTT (s)	41.0(37.6–44.8)	41.8(37.9–46.4)	0.184
TT (s)	14.8(14.2–15.4)	14.7(14.1–15.3)	0.234
FIB (g/L)	5.5(4.4–6.4)	5.9(4.9–7.0)	0.004
D-Dimer (μg/ml)	1.0(0.6–1.8)	1.4(0.8–2.3)	0.002

*ANC1* absolute neutrophil count after IVIG treatment. Values are expressed as Median (interquartile range) / Number (percentage). Continuous variables were compared using Kruskal-Wallis test, categorical variables were compared using Chi-square test

### Comparison of treatment protocols between KD patients with and without neutropenia

We compared differences in IVIG treatment duration, IVIG dosage, use of dipyridamole, incidence of CAL after treatment, incidence of IVIG non-responders and gender between NKD and NNKD groups. We found that (1) IVIG treatment duration differed between the two groups, being longer in the NKD than the NNKD group (P = 0.002); (2) the incidence of neutropenia in children treated with the 2 g/kg*1d scheme was lower than in those treated with 1 g/kg*2d (*P* = 0.009); (3) in patients followed up with UCG for 3 months after IVIG treatment, the incidence of CAL was higher in the NKD group than in the NNKD group (*P* = 0.008); and (4) the probability of male patients with neutropenia in the NKD was higher than that in the NNKD group, but there’s no sex differences between groups (*P* = 0.715) (Table 2).

**Table 2:** Treatment and outcome of children with KD

Group	NKD	NNKD	*P* value
IVIG treatment duration (days)	7(6–8)	6(6–7)	0.002
IVIG dosage			0.009
2d/kg*1d	77(14.3%)	462(85.7%)	
1 g/kg*2d	43(22.4%)	149(77.6%)	
dipyridamole	39(37.5%)	224(33.3%)	0.404
CAL after treatment^a^	58(31.9%)	250(22.8%)	0.008
CAA after treatment	4(6.9%)	26(10.4%)	0.418
IVIG nonresponders	5(3.0%)	51(5.8%)	0.167
Gender			0.715
Female	63(32.0%)	389(33.3%)	
Male	134(68.0%)	779(66.7%)	

^a^CAL after treatment is defined as patients with persistent CAL after IVIG treatment. Values are expressed as Median (interquartile range) / Number (percentage). Continuous variables were compared using Kruskal-Wallis test, categorical variables were compared using Chi-square test

### Subgroup analysis of CAL between KD patients with and without neutropenia

As mentioned above, patients followed up with UCG for 3 months after IVIG treatment, the incidence of CAL was higher in the NKD group than in the NNKD group (P = 0.008). Then we performed statistical analysis of the 3 subgroups according to the standard of CAL. As shown in Table 3, we found that the smaller the age, the greater the probability of CAL, regardless of whether there is neutropenia in children with KD. The incidence of CAL in NKD group was higher than NNKD group in children with KD less than 3 years of age, but there was no statistical difference (*P* = 0..110).

**Table 3:** Subgroup analysis of KD patients with CAL

Group	NKD	NNKD	*P* value
CAL	58(31.9%)	250(22.8%)	0.008
< 3 (years)	54(93.1%)	213(85.2%)	0.110
3–9(years)	4(6.9%)	35(14.0%)	0.143
> 9(years)	0	2(0.8%)	–

Values are expressed as Number (percentage). Categorical variables were compared using Chi-square test

### Comparison of the proportion of CAA in the NKD and NNKD

There are 30 patients developed CAA followed up with UCG for 3 months after IVIG treatment. The incidence of CAA in NKD was lower than NNKD, but there was no statistical difference. CAA was divided into small, medium and giant according to the size of the internal diameter, the proportion of each of the two groups was shown in Table 4. Comparison of the incidence of CAA among small and medium, medium and giant, small and giant, with no statistical significance (*P* = 0.131, *P* = 0.308 and *P* = 0.656, respectively).

**Table 4:** The proportion of CAA in patients with KD

Group	NKD	NNKD	*P* value
CAA	4(13.3%)	26(86.7%)	0.418
Small CAA	1(6.3%)	15(93.7%)	0.131^a^
Medium CAA	3(27.3%)	8(72.7%)	0.308^b^
Giant CAA	0	3(100%)	0.656^c^

Values are expressed as Number (percentage). Categorical variables were compared using Chi-square test. a. statistical results between small CAA and medium CAA; b. statistical results between medium CAA and giant CAA; c. statistical results between small CAA and giant CAA

### Comparison of clinical manifestations between KD patients with and without neutropenia

Table 5 shows the incidence of five common clinical manifestations among children with and without neutropenic KD. There are no statistically significant differences between groups in the incidence of rash, conjunctivitis, changes in lips, and changes in extremities. However, the incidence of cervical lymphadenopathy was significantly higher in the NNKD group (*P* < 0.001).

**Table 5:** Clinical manifestations of KD in children

Group	NKD	NNKD	*P* value
rash	145(73.6%)	865(74.1%)	0.893
conjunctivitis	172(87.3%)	1005(86.0%)	0.634
changes in lips	177(89.8%)	1087(93.1%)	0.111
changes in extremities	139(70 .6%)	867(74.2%)	0.279
cervical lymphadenopathy	95(48.2%)	747(64.0%)	< 0.001

Values are expressed as Number (percentage). Categorical variables were compared using Chi-square test

### The correlation between the degree of reduction in granulocyte count and CAL

To analyze the relationship between reduced granulocyte count and CAL, we first performed a logistic regression analysis using △ANC as the continuous variable and CAL within 3 months after treatment as the dependent variable. We found no significant correlation between the degree of reduction in granulocytes and the risk of CAL within 3 months after treatment, even after controlling for age and sex.

Secondly, we performed a logistic regression analysis using five categories of △ANC as the continuous variable and CAL within 3 months after treatment as the dependent variable. We found that the risk of CAL was lowest when the absolute reduction in granulocytes was between 5.653 to 7.850 (OR = 0.768; 0.507, 1.165). This was true even after controlling for age and sex (OR = 0.760; 0.499, 1.158) (Table 6).

**Table 6:** The independent effect of △ANC on the risk of CAL among KD patients

Exposure	Non-adjusted	Adjusted
△ANC (× 10^9^/L)	1.000(0.989,1.012)0.935	1.000(0.989,1.011)0.984
△ANC(×10^9^/L)(equal percentiles)		
≤ 3.015	1.0	1.0
3.016–5.652	0.879(0.585,1.320)0.534	0.873(0.578,1.319)0.519
5.653–7.850	0.768(0.507,1.165)0.214	0.760(0.499,1.158)0.202
7.851–11.474	1.042(0.700,1.552)0.839	1.021(0.682,1.528)0.919
≥ 11.475	1.091(0.734,1.621)0.667	1.138(0.762,1.702)0.527

**Fig. 1:**
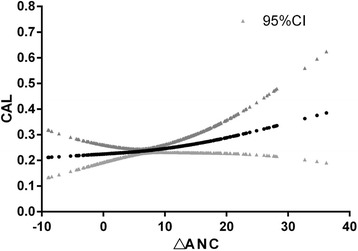
Threshold effect analysis takes the break point (k) as 6. When k < 6 the OR = 0.97 (0.91, 1.03) and *p* = 0.3164; when k > 6, the OR = 1.04 (1.00, 1.07) and *p* = 0.0323

Values are expressed as OR (95%CI) P value;

Adjusted model: age (months); gender;

Finally, we generated a curve using △ANC as the continuous variable and CAL within 3 months after treatment as the dependent variable (Fig. 1). Threshold effect analysis identified the break point as 6 (△ANC = 6 × 10^9^/L). We found a correlation between the degree of reduction in granulocytes and CAL when the break point is greater than 6, with a higher rate of CAL in patients with a greater reduction in granulocytes (*P* = 0.0323).

## Discussion

KD is a systemic vasculitis that presents as an acute febrile illness. CAL is the main complication of this disease, and its incidence can be reduced by high-dose IVIG treatment, which acts to reduce inflammation [6]. In our practice, we have found that KD patients treated with IVIG often have reduced neutrophil counts during follow up, and some even developed agranulocytosis. In this study, patients were divided into two groups for statistical analysis, and we found that the incidence of neutropenia after IVIG treatment was related to the IVIG dosage protocol. Namely, we found that the 2 g/kg*1d scheme was associated with a reduced incidence of neutropenia compared to the 1 g/kg*2d scheme. Furthermore, at the 3-month follow-up, we found that there was a statistically significant difference in the incidence of CAL between groups, which was higher in patients with NKD. Then we performed a subgroup analysis of the different age groups according to the CAL criteria. It was found that the incidence of CAL in NKD group higher than NNKD group in children with KD less than 3 years of age, but there was no statistical significance. Similarly, there were no statistically significant differences in the incidence of CAL among the subgroups.

CAL is the most common complication of KD and is associated with fever duration [7–9], vascular endothelial growth factor [10, 11], B-type natriuretic peptide [12], serum albumin [13], serum sodium [14], CRP [15], platelet-neutrophil aggregates [6], and inflammatory cytokines including tumor necrosis factor-α and inter-leukin-6 [15, 16]. In this study, we found that some patients developed neutropenia after IVIG treatment. These patients were followed up with UCG at 3 months, and we identified a higher incidence of CAL in patients who developed neutropenia after treatment. The curve fitting analysis of the degree of reduction in granulocytes and CAL shows that when the breaking point is 6 (△ANC = 6 × 10^9^/L); that is, the rate of CAL is higher when the degree of reduction in granulocytes is greater. Therefore, children who develop neutropenia after IVIG treatment should be followed up with regular UCG in order to facilitate the early detection and treatment of CAL, and this is especially important in children with a significant reduction in granulocytes.

KD is an inflammatory disease and neutrophils are important mediators involved in the inflammatory response. Consistent with the results described in this study, Tsujimoto et al [17] found that treatment with IVIG resulted in a significant reduced in neutrophil counts. The mechanisms for this observation have not been clearly elucidated, but we propose several plausible explanations. First, in our study, 30–50 mg/kg aspirin therapy was used in children with KD on admission, drawing blood from the vein when defervescence after 3 days and aspirin did not decrease at the same time, therefore, aspirin induced neutropenia is not considered. IVIG is another effective drug for the treatment of KD, and it has been reported that IVIG can induce neutrophil apoptosis and degranulation in vitro [18]. IVIG inhibits the activated immune system, lowers the levels of inflammatory factors, and reduces the production of cytokines, thereby reducing the inhibition of neutrophil apoptosis. IVIG mainly acts through the Fas pathway and the caspase pathway. IVIG contains Fas antibody which contributes to apoptosis by activating the intracellular caspase system after binding to the Fas antigen on neutrophils and monocytes [4]. Second, KD is an autoimmune disease characterized by elevated neutrophil counts in the acute phase, with neutrophil destruction by autoantibodies during convalescence [5]. Third, the results of this study show that the level of WBC and neutrophils in children with neutropenia before IVIG treatment is lower than in those without neutropenia, and therefore it is possible that the neutropenia after treatment may be related to the basal neutrophil count at the time of disease onset.

This study is strengthened by its large sample size. However, there are certain limitations worth noting. First, our study is a single center study and therefore further multicenter studies are warranted in order to assess the generalizability of these findings. Second, the results may lack some accuracy due to the small sample of patients included in the IVIG dosage sub-analysis.

## Conclusions

Neutropenia is an important complication in children with KD treated with IVIG, and is less likely among those treated with 2 g/kg*1d IVIG. The results of UCG follow-up showed that the probability of CAL was higher in patients with neutropenic KD compared to non-neutropenic KD, and in patients with a greater reduction in granulocyte counts. Therefore, children with KD should be treated with 2 g/kg*1d IVIG and monitored to prevent a large degree of reduction in granulocytes (△ANC ≥ 6 × 10^9^/L). Early diagnosis and treatment of CAL is essential to maximizing outcomes in this patient population.

## Abbreviations

ALB: Albumin
ALT: Alanine transaminase
ANC: Absolute neutrophil count
APTT: Activated partial thromboplastin time
BNP: Brain natriuretic peptide
CAA: Coronary artery aneurysm
CAL: Coronary artery lesions
CRP: C-Reactive protein
ESR: erythrocyte sedimentation rate
FIB: Fibrinogen
Hb: Hemoglobin
IVIG: Intravenous immune globulin
KD: Kawasaki disease
Na: Sodium
PLT: Platelets
PT: Prothrombin time
TT: Thromboplastin time
UCG: Ultrasonic echocardiography
WBC: White blood cell
